# Elevating Skincare Science: Grape Seed Extract Encapsulation for Dermatological Care

**DOI:** 10.3390/molecules29163717

**Published:** 2024-08-06

**Authors:** Maria Leonor Castro, João Azevedo-Silva, Diana Valente, Adriana Machado, Tânia Ribeiro, João Paulo Ferreira, Manuela Pintado, Oscar L. Ramos, Sandra Borges, Sara Baptista-Silva

**Affiliations:** 1 CBQF—Centro de Biotecnologia e Química Fina—Laboratório Associado, Escola Superior de Biotecnologia, Universidade Católica Portuguesa, Rua Diogo Botelho 1327, 4169-005 Porto, Portugal; marialeonorcastro13@gmail.com (M.L.C.); jasilva@ucp.pt (J.A.-S.); dvalente@ucp.pt (D.V.); armachado@ucp.pt (A.M.); tribeiro@ucp.pt (T.R.); jpferreira@ucp.pt (J.P.F.); mpintado@ucp.pt (M.P.); oramos@ucp.pt (O.L.R.); sandraferreiraborges@gmail.com (S.B.); 2 Amyris Bio Products Portugal, Unipessoal Lda, Rua Diogo Botelho 1327, 4169-005 Porto, Portugal; 3 Associação CECOLAB, Laboratório Colaborativo para a Economia Circular, Centro Empresarial, Rua Nossa Senhora da Conceição, 2, Oliveira do Hospital, 3405-155 Coimbra, Portugal

**Keywords:** grape by-products, phenolic compounds, microdispersions, sustainability, skincare

## Abstract

The skin is the largest organ in the human body and serves multiple functions such as barrier protection and thermoregulation. The maintenance of its integrity and healthy structure is of paramount importance. Accordingly, technological advances in cosmetic sciences have been directed towards optimizing these factors. Plant-derived ingredients have been explored for their bioactivity profiles and sustainable sources. Grape by-products contain a group of bioactive molecules that display important biological activities. Nonetheless, many of these molecules (e.g., phenolic compounds) are unstable and susceptible to degradation. So, their encapsulation using nano/microsystems (i.e., microdispersions) has been explored as a promising solution. In this work, two grape seed extracts were obtained, one from a single grape variety (GSE-Ov) and another from a mix of five grape varieties (GSE-Sv). These extracts were analysed for their antioxidant and antimicrobial activities, as well as their chemical composition and molecular structure. The extract that showed the most promising properties was GSE-Ov with a DPPH IC_50_ of 0.079 mg mL^−1^. This extract was encapsulated in soy lecithin microdispersions coated with pectin, with an encapsulation efficiency of 88.8%. They showed an in vitro release of polyphenols of 59.4% during 24 h. The particles displayed a zeta potential of −20.3 mV and an average diameter of 13.6 µm. Microdispersions proved to be safe under 5 and 2.5 mg mL^−1^ in HaCaT and HDF cell models, respectively. Additionally, they demonstrated anti-inflammatory activity against IL-1α when tested at 2 mg mL^−1^. This work enabled the valorisation of a by-product from the wine industry by using natural extracts in skincare products.

## 1. Introduction

The skin is the largest organ of the human body, covering approximately 2 m^2^ [1]. It comprises functions such as protection against trauma, solar radiation, toxins, and infections; preservation of water and electrolytes; thermoregulation; and storage of water, vitamin D, and fat [1,2]. Over time, the skin is subjected to ageing, which is induced by both extrinsic/environmental factors (e.g., solar radiation, pollution) and intrinsic/chronological ones (e.g., cellular senescence, increased expression of skin-degrading enzymes), all leading to reduced structural integrity and loss of physiological function [3,4]. Aside from ageing, the skin is also susceptible to a variety of disorders that have been mainly associated with inflammatory states and skin microbiota dysbiosis [5]. For example, diseases such as acne vulgaris, atopic dermatitis, and psoriasis, which are characterized by inflammatory manifestations (e.g., papules, pustules, flares, peeling, etc.), have been related to the imbalance of skin microorganisms mainly belonging to the *Cutibacterium*, *Corynebacterium*, and *Staphylococcus* genera [6,7,8,9,10,11,12].

In this regard, to preserve skin health, plant-derived ingredients have gained interest in the last few years. Grapes are one of the world’s biggest fruit crops, with approximately 80% of its production being destined for winemaking [13,14]. High levels of organic waste are produced during the winemaking process, being primarily composed of grape pomaces (62%), lees (14%), stalks (12%), and dewatered sludge [15], reaching approximately 14.5 million tons per year in Europe [16]. For instance, 25% of grape pomace is seed [17], which, as an agro-industrial by-product, may be responsible for environmental problems when improperly managed [15,18] because of the low pH and high concentration of phenolic compounds, which can resist biological degradation [15]. Notwithstanding, it is possible to valorise these wastes effectively by extracting their bioactive compounds, especially the above-mentioned phenolic molecules [15,18]. Phenolic compounds are secondary plant metabolites, and, in grapevines, they span from structural cell wall components, such as lignin and tannins, to specialized ones, such as phenolic acids and flavonoids. Phenolic compounds are known to have several properties such as antioxidant, anti-inflammatory, antibacterial, anticarcinogenic, antidiabetic, and cardioprotective effects, exhibiting great potential for several sectors, particularly the cosmetic industry [19,20,21].

In recent years, the delivery of grape by-product-derived compounds in micro- or nanocarriers has been pursued as an innovative and effective strategy to boost their stability, bioavailability, and biological activity at the target locations [22]. In particular, micro-scaled encapsulation does not raise some of the concerns about nanostructures (e.g., bioaccumulation, penetration of cellular and subcellular structures, regulation [23,24,25]); it allows for a better appearance and a smoother touch of a product, the encapsulation of high-molecular-weight compounds, and greater stability over time with less ability to aggregate, and sometimes, it presents more automated, simple, and fast manufacturing processes (since size is not a restrictive criterion) [26]. Among the several carriers designed for different activities (reviewed in Castro et al. [23]), particularly tailored for skin delivery, microdispersions have been the favoured option since they protect encapsulated compounds, prevent their degradation, and are able to transport and release them more effectively on the targeted layers of the skin because of their lipidic character. On their own, microdispersions can also promote the repair of the skin protective barrier [27,28].

Thus, the main goal of this work was to obtain, develop and characterize a grape seed extract for potential skincare and cosmetic applications. In addition, we aimed to employ an encapsulation method that improves extract stability, provides controlled delivery, and has high overall efficacy. Moreover, this research proposed a sustainable approach to a common by-product, thus promoting a circular economy.

## 2. Materials and Methods

### 2.1. Biomass

The seeds were from red grapes grown in the Alto Douro region of Portugal and were provided by Symington Family Estates (Vila Nova de Gaia, Portugal), a table wine and Port wine company. In this study, the following types of red grape seeds were used: (i) seeds from one wine grape variety commonly used for table wine (GSE-Ov) and (ii) a mix of seeds from five selected grape varieties, commonly used for Port wine (GSE-Sv). Ethanol absolute anhydrous solvent was purchased from Carlo Erba Reagents S.A.S. (Val-de-Reuil, France). All the remaining reagents were analytical grade or higher, as described below.

### 2.2. Preparation of Grape Seed Extracts (GSEs)

**Figure 1:**
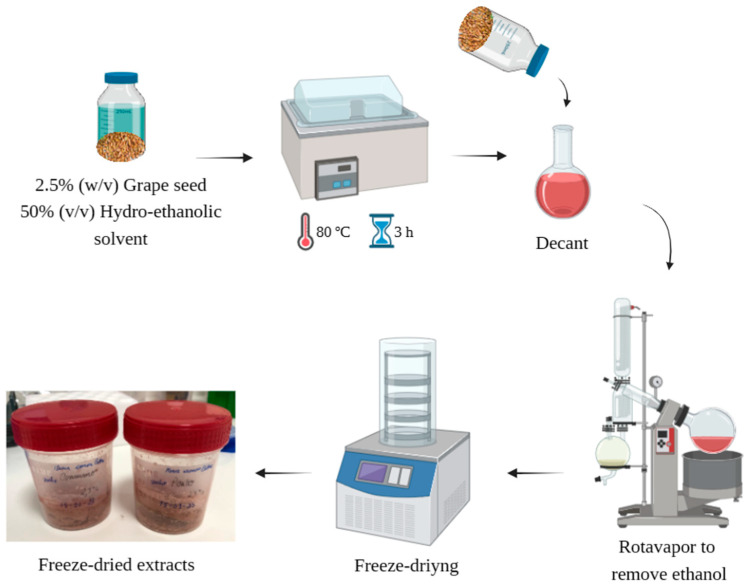
Schematic representation of the extraction of the GSEs. Created with BioRender.com.

The GSE was obtained as described by Bucić-Kojić et al. [29], with some modifications. Briefly, milled grape seeds were subjected to a solid–liquid extraction process using a hydro-ethanolic solvent (1:1 (*v v*^−1^)) at a 1:40 (*w v*^−1^) ratio. The process was carried out in a shaking water bath (Shaking Bath UNITRONIC VAIVÉN, JP SELECTA, Barcelona, Spain) for 3 h at 80 °C. After extraction, the mixture was decanted for solid–liquid separation, and the ethanol in the supernatant was evaporated using an R-210 Rotavapor System (Rotary Evaporator BÜCHI R-210, Flawil, Switzerland). Finally, the resultant supernatant was frozen at −80 °C and then lyophilized (GAMMA 2–16 LSC plus, Christ, Osterode am Harz, Germany). A schematic representation of the extraction process is shown in Figure 1.

### 2.3. Antioxidant Activity

The antioxidant activity of both GSEs (GSE-Ov and GSE-Sv) was assessed using the following methods: the 2,2′-azinobis (3-ethylbenzothiazoline-6-sulfonic acid) (ABTS) assay, the 2,2-diphenyl-1-picrylhydrazyl (DPPH) assay, and the Folin–Ciocalteau method. For these methods, freeze-dried GSEs were weighed and solubilized in methanol.

#### 2.3.1. ABTS Radical Cation Decolorization Assay

The ABTS assay was performed according to Gonçalves et al. [30]. An ABTS^+^ stock solution was prepared by mixing two aqueous solutions (ultrapure water), one of ABTS with a concentration of 7 mM and the other of potassium persulfate (K_2_O_8_S_2_) with a concentration of 2.44 mM. The resulting solution was left to stir for approximately 16 h, covered with aluminium foil, and then stored at 4 °C for a maximum of 1 month. An ABTS^+^ working solution was made by filtering the prior with a 0.45 µm syringe filter and diluting it with water until the absorbance at 734 nm was 0.70 ± 0.02. Also, a Trolox solution was prepared with a 1000 µM concentration and stored at −20 °C. For this solution, Trolox was solubilized in methanol and the volume was completed with a PBS solution (75 mM NaH_2_PO_4_), pH 7.4. Briefly, for the assay procedure, in each well of a 96-well microplate, 20 µL of the sample (each dilution), Trolox, or solvent (methanol) for the control, were pipetted in triplicate, followed by 180 µL of ABTS^+^ working solution. After incubation for 6 min at 30 °C, the absorbances were measured at 734 nm using a microplate reader (Microplate Reader Biotek Synergy H1, Santa Clara, CA, USA). The scavenging activity (SA) of the samples was determined using Equation (1), and the results were expressed as IC_50_ milligrams per gram of dry extract (mg g^−1^ dry extract).

(1) $$SA\;\left(\%\right)=\left[\frac{{Abs}_{CTL}-{Abs}_{SPL}}{{Abs}_{CTL}}\right]\times 100\;$$

where *Abs_CTL_* is the absorbance of the control and *Abs _SPL_* is the absorbance of the samples.

#### 2.3.2. DPPH Radical Cation Decolorization Assay

The DPPH assay was performed according to Brand-Williams et al. [31]. First, a 100% methanolic DPPH stock solution was prepared with a 600 µM concentration and then stored protected from light at −20 °C. From the previous one, a DPPH working solution was prepared (by dilution with methanol) until its absorbance was 0.600 ± 0.100 at 515 nm. Also, a Trolox solution was prepared in methanol with a 1000 µM concentration and stored at −20 °C. Briefly, in each well of a 96-well microplate, 25 µL of sample (each dilution), Trolox, or solvent (methanol) used as the control was pipetted in triplicate, followed by 175 µL of the DPPH working solution. After incubation for 30 min at 25 °C, the absorbance was measured at 515 nm using a microplate reader. The scavenging activity (SA) of the samples was also determined using Equation (1), and the results were expressed as IC_50_ milligrams per gram of dry extract (mg g^−1^ dry extract).

#### 2.3.3. Folin–Ciocalteau Colorimetric Method

The total phenolic content of the GSE was determined by the Folin–Ciocalteau method with some modifications [32]. First, a 7.4% (*w v*^−1^) Na_2_CO_3_ solution was prepared in deionized water (dH_2_O). Also, a Folin–Ciocalteau working solution was prepared by diluting 2 mL of Folin–Ciocalteau reagent (Sigma Aldrich, St. Louis, MO, USA) to 10 mL with dH_2_O. This solution was prepared daily when needed. Furthermore, a gallic acid standard solution with a concentration of 1 mg mL^−1^ was prepared in methanol and stored at −20 °C. Briefly, for the procedure, in each well of a 96-well microplate, 30 μL of sample (each dilution), gallic acid, or solvent (methanol) for the blank was pipetted in triplicate, followed by 100 μL of Folin–Ciocalteau working solution and 100 μL of 7.4% Na_2_CO_3_, in that order. After incubation for 30 min at 25 °C, the absorbance was measured at 765 nm using a microplate reader. The total phenolics content of the samples was determined by interpolation with a gallic acid calibration curve (0.025–0.200 mg mL^−1^) and expressed as milligrams of gallic acid equivalent per gram of dry extract (mg GAE g^−1^ dry extract).

### 2.4. Antimicrobial Activity

For the evaluation of the GSE-Ov and GSE-Sv antimicrobial activity, several microbial-relevant strains usually present in the skin microbiota were used including *S. epidermidis* (DSM 20044), methicillin-susceptible *S. aureus* (MSSA) (ATCC 29213), methicillin-resistant *S. aureus* (MRSA) (DSM 11729), and *C. acnes* (DSM 1897) (obtained from the collection culture of CBQF, Universidade Católica Portuguesa). The method was performed as described by Pinela et al. [33], with modifications. Colonies of the microbial strains were transferred and cultured overnight on Mueller Hinton (MH) agar (Biokar, Allonne, France) plates at 37 °C for 24 h. *C. acnes* was grown on Tryptic Soy (TS) agar (Biokar) supplemented with 5% sheep blood (Thermo Fisher Scientific, Waltham, MA, USA), with an anaerobic atmosphere controlled at 37 °C for 48 h. After incubation, the inoculum suspensions were adjusted to a turbidity of 0.5 McFarland (equal to 1.5 × 10^8^ colony-forming units (CFUs) mL^−1^)) in sterile saline solution. For antimicrobial activity, 100 µL GSEs (100 mg mL^−1^) were mixed with 100 µL media and serially diluted in the same media. Then, 100 μL of inoculant was added to each GSE dilution and incubated for ca. 24 h at 37 °C. After incubation, all samples were plated using the drop technique [34] on MH agar or TS agar supplemented with blood and then incubated for ca. 24 h or 48 h at 37 °C, for MBC (minimum bactericidal concentration) determination. Culture media with and without inoculum were used as controls.

### 2.5. Fourier-Transform Infrared (FTIR) Analysis

An FTIR analysis of the GSEs was carried out using a Spectrum 100 FTIR spectrometer with a horizontal attenuated total reflectance sample accessory (PIKE Technologies, Fitchburg, WI, USA) and a diamond/ZnSe crystal. Horizon MBTM FTIR software, version 10.7.2 was used. All spectra were collected with 16 scans and a 4 cm^−1^ resolution in the 4500–500 cm^−1^ wavenumber interval. In addition, baseline, point adjustment, and spectra normalization were performed. All samples were analysed in triplicate.

### 2.6. GSEs Composition Analysis by High-Performance Liquid Chromatography (HPLC)

The GSEs were precisely weighed, dissolved in hydro-ethanolic solvent (1:1) at a final concentration of 10 mg mL^−1^, and then filtered into vials using 0.45 μm syringe filters. For the composition analysis, samples were analysed on a Waters Alliance e2695 Separate Module HPLC coupled to a C_18_ Phenomenex column (250 × 4.6 mm × 5 μm, Kromasil) and a Diode Array Detector (DAD), according to Ribeiro et al. [35]. Detection was achieved at wavelengths of 280 (derivates of hydroxybenzoic acids), 320 (derivatives of hydroxycinnamic acids), 360 (flavonols), and 528 nm (anthocyanins). The mobile phase was composed of (A) acetonitrile/water (5:95 *v v*^−1^) 0.1% Trifluoroacetic acid (TFA) and (B) acetonitrile 0.1% TFA. The flow rate was set at 1.0 mL min^−1^, and the injection volume was 20 μL. Data acquisition and analysis were accomplished with Software Empower 3. The identification was performed by comparing the retention time and absorbance spectra with pure standards.

### 2.7. GSE-Ov Encapsulation with Microdispersions

**Figure 2:**
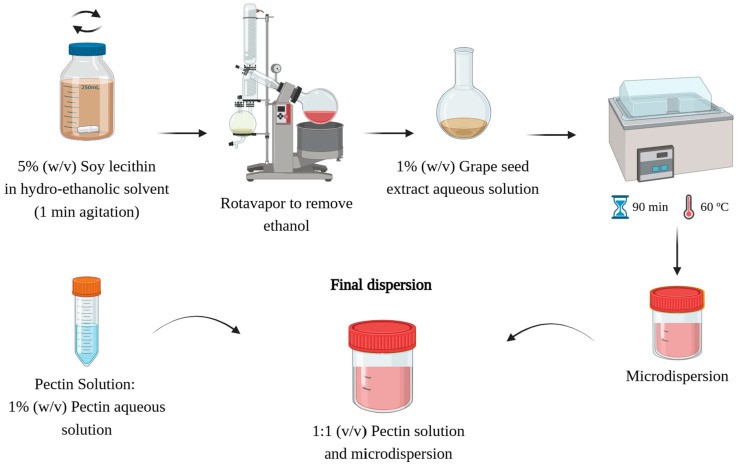
Schematic representation of the encapsulation process. Created with BioRender.com.

Microdispersions were prepared by the lipid film hydration method, as described by Machado et al. [36]. Briefly, lecithin (Alfa Aesar, Haverhill, MA, USA) was solubilized in absolute ethanol at a 1:20 (*w/v*) ratio, and the mixture was stirred for 1 min. Then, an R-210 Rotavapor System (Rotary Evaporator BÜCHI R-210, Flawil, Switzerland) was used to evaporate the ethanol until a lipid film was observed on the flask walls. Then, 0.2 g of GSE-Ov and 20 mL of dH_2_O (for a loaded microdispersion), or just 20 mL of dH_2_O (for an empty microdispersion), were added to the flask, and the mixture was left in a shaking water bath at 60 °C for 90 min. Finally, 1% (*w v*^−1^) pectin aqueous solution was mixed with the microdispersion in a 1:1 (*v v*^−1^) ratio to enhance its stability (adjust the electrical charge). A schematic representation of the encapsulation process is shown in Figure 2.

### 2.8. Microdispersion Characterization

#### 2.8.1. Zeta Potential

For the microdispersion characterization, measurements of zeta potential were performed at room temperature (25 °C) in a folded capillary cell using a Zetasizer Nano ZS (Malvern Instruments Ltd., Malvern, UK) with a He-Ne laser wavelength of 633 nm and a detection angle of 173°. The analysis was performed in triplicate, and the results were expressed as average ± standard deviation (mV).

#### 2.8.2. Particle Size Distribution

For particle size distribution assessment, we used a Malvern Mastersizer 3000—Laser Diffraction (Malvern Instruments Ltd.) with a refractive index of 1.457 and an absorption index of 0.01 parameters selected. dH_2_O was used as a dispersant. According to the laser diffraction through the particles of the material, a scattering pattern was generated and used to calculate the particle size via Mie theory. Both the empty and loaded microdispersions were analysed six times, and the data were presented as the mean of all measurements.

#### 2.8.3. Morphology

Microdispersion morphology was assessed using a Pro Scanning Electron Microscope (Thermo Scientific™, Waltham, MA, USA). Before the procedure, the microdispersion samples (empty microdispersion) were freeze-dried and analysed in splinters and powder forms. For sample preparation, they were placed onto metal plates and coated with a thin gold layer using a Sputter Coater (Polaron, Bad Schwalbach, Germany) under vacuum. SEM was used with the magnifications of 500, 1000, 2000, and 4000× and with an electron beam of 10 kV. The images presented are representative of the morphology of the sample.

#### 2.8.4. FTIR Analysis

An FTIR analysis of the encapsulated GSE-Ov and encapsulant was carried out as previously described in Section 2.5.

#### 2.8.5. Encapsulation Efficiency

The microdispersion’s encapsulation efficiency (EE) was assessed by an indirect method, as follows. After GSE encapsulation, the supernatant was recovered and analysed by the Folin–Ciocalteau method for total phenolic content determination, as described in Section 2.3.3. EE was calculated by the difference between the total phenolic content of the extract and the total phenolic content of the supernatant after encapsulation, according to Equation (2).

(2) $$EE\;\left(\%\right)=\left[\frac{{TPC}_{GSE}-{TPC}_{SN}}{{TPC}_{GSE}}\right]\times 100$$

where *TPC_GSE_* is the total phenolic content of the GSE and *TPC_SN_* is the total phenolic content of the supernatant after encapsulation. This analysis was performed in triplicate.

#### 2.8.6. In Vitro Phenolic Compounds Release Assay

The release profile of phenolic compounds by the microdispersion was assessed as follows. After GSE encapsulation, the mixture was centrifuged (5000 rpm, 9 min; Hettich Zentrifugen, Tuttlingen, Germany), and a sample was retrieved for initial total phenolic content determination (0 h). Then, the supernatant was removed and replaced with 5 mL PBS at pH 5.4 to mimic the physiological conditions of the skin. Then, 500 µL samples were taken at the following times: 1, 2, 4, 6, and 24 h. Immediately after collecting the samples, the retrieved volume was replaced with PBS for volume maintenance. Finally, all samples were centrifuged (13,220 rpm, 5 min; Andreas Hettich GmbH & Co. KG, Tuttlingen, Germany) and analysed using the Folin–Ciocalteau method as previously described in Section 2.3.3. During the experiment, the mixture was kept under stirring at 100 rpm and 37 °C, and the analysis was performed in triplicate.

### 2.9. Cell Culture

Immortalized human keratinocytes (HaCaT) were obtained from CLS (300493) and human dermal fibroblast (HDF) were obtained from ATCC (PCS-201-012). The cells were cultured at 37 °C in a humidified atmosphere of 95% air and 5% CO_2_ as monolayers using Dulbecco’s Modified Eagle Medium (DMEM, Gibco, Thermo Fisher Scientific), supplemented with 10% (*v v*^−1^) foetal bovine serum (FBS, Gibco, Thermo Fisher Scientific) and 1% (*v v*^−1^) antibiotic/antimycotic. HaCaT cells were used between passages 52 and 56, and HDF between passages 4 and 6.

### 2.10. Cytotoxicity

The cytotoxicity of GSE-Ov, encapsulated GSE-Ov, and the encapsulant was assessed through the PrestoBlue™ Cell Viability test (Thermo Fisher Scientific), as specified by the manufacturer. HaCaT and HDF cells were seeded at 1.0 × 10^5^ cells mL^−1^ in 96 well microplates and left to adhere in incubation overnight. Then, the cells were exposed to GSE-Ov or encapsulated GSE-Ov in serial dilutions starting at 10 mg mL^−1^, or encapsulant at 10 mg mL^−1^, for 24 h at 37 °C with 5% CO_2_ in a humidified environment using DMEM. Concisely, 90 µL of the sample and culture medium for the positive control or 10% dimethyl sulfoxide (DMSO, Sigma Aldrich) for the negative control were added to each well. In two separate studies, each sample was evaluated in quadruplicate.

After incubation, 10 µL of PrestoBlue™ Cell Viability Reagent was added to each well of the plate and left to incubate, protected from light, for approximately 2 h in a humidified atmosphere at 37 °C and 5% CO_2_. Lastly, fluorescence was measured using a microplate reader. The results were presented in percentage of metabolic inhibition in comparison to the positive control, according to the ISO 10993-5 standard [37], where an inhibition of more than 30% is deemed cytotoxic.

### 2.11. Quantification of Pro-Inflammatory Cytokines

HaCaT cells were seeded at 2.5 × 10^5^ cells mL^−1^ in 12 well (1 mL per well) microplates for 24 h. Then, the cells were exposed to GSE-Ov, encapsulated GSE-Ov, or the encapsulant at the following concentrations: 0.1 and 0.025 mg mL^−1^, 2 and 0.5 mg mL^−1^, and 10 mg mL^−1^, respectively. The microplate was left to incubate for 24 h in the conditions mentioned above. Briefly, the culture medium was used as a control, and SRM 1648 Urban Particulate Matter obtained from the NIST (Gaithersburg, ML, USA) was used as an inflammatory stimulus. Each sample was evaluated in duplicate, in two independent studies.

After 24 h, the supernatants of each well were collected to quantify the level of proinflammatory cytokines IL-1α IL-6 using the respective ELISA MAX™ Deluxe Set Human kit (BioLegend, San Diego, CA, USA), according to the manufacturer’s instructions.

Then, the cell total protein was quantified using the Pierce™ BCA Protein Assay Kit (Thermo Fisher Scientific), as recommended by the manufacturer. Finally, the absorbance was measured at the wavelengths of 450 and 570 nm for IL-1α and 562 nm for IL-6 using a microplate reader (Microplate Reader Biotek Synergy H1, USA). The final results were presented in pg of cytokine mg^−1^ of total protein.

### 2.12. Human Pro-Collagen I α1 Quantification

HDF cells were seeded at 3 × 10^5^ cells mL^−1^ in 12 well (1 mL per well) microplates for 24 h. After that, the cells were exposed to GSE-Ov, encapsulated GSE-Ov, or the encapsulant at the following concentrations: 0.1 and 0.025 mg mL^−1^, 2 and 0.5 mg mL^−1^, and 10 mg mL^−1^, respectively. Culture medium was used as a control. In two separate studies, each sample was evaluated in duplicate. Total protein was quantified as previously indicated.

The quantification of collagen I α1 was performed using the Human Pro-Collagen 1 alpha 1 CatchPoint^®^ SimpleStep ELISA^®^ Kit (ABCAM, ab229389), as recommended by the manufacturer. The total protein used was 100 ng. The fluorescence was measured at Ex/Cutoff/Em 530/570/590 nm using a microplate reader. The final results were expressed in pg of collagen μg^−1^ of total protein.

### 2.13. Statistical Analysis

The IBM^®^ SPSS^®^ Statistics 26 program was utilized for statistical analysis. The data were first checked for normality (the Shapiro–Wilk test, n < 50, or the Kolmogorov–Smirnov test, n > 50). To assess differences between more than two groups, a one-way ANOVA test (normal distribution) with Tukey’s HSD post hoc test or a Kruskal–Wallis test (non-normal distribution) was used. A Student’s *t*-test (normal distribution) or a Mann–Whitney test (non-normal distribution) was used to compare two groups. The significance level was set at 0.05.

## 3. Results and Discussion

### 3.1. Initial Screening of GSE Potential and Characterization

#### 3.1.1. Antioxidant Activity

The antioxidant activity of both GSEs was evaluated using the ABTS and DPPH assays, and the results are presented in Table 1. For both methods, GSE-Ov presented higher antioxidant activity (i.e., lower IC_50_—half-maximal inhibitory concentration). For this extract, the obtained values were 0.138 and 0.079 mg mL^−1^ for ABTS and DPPH, respectively, whereas for GSE-Sv, the obtained values were 0.163 and 0.088 mg mL^−1^, respectively. Grape species, individual cultivars, cultivation conditions, maturation stages, and seasonal fluctuations, according to Mandić et al. [38], may impact phenolic biosynthesis and the antioxidant capacity of grape seeds, which can be a possible explanation for the obtained differences between GSEs. GSE-Ov presented a higher total phenolic content (258.96 ± 30.94 mg GA eq g^−1^ dry extract) than GSE-Sv (203.72 ± 42.64 mg GA eq g^−1^ dry extract), which is in accordance with the antioxidant activity results. Comparing both methods, the DPPH assay allowed us to obtain higher antioxidant activity for both GSEs. This is due to the nature of the assays themselves, once the samples were prepared in methanol; therefore, only in a methanol-like solvent (like the one used for DPPH) can they present proper antioxidant activity [5,39].

By comparing the obtained results with the antioxidant activity of two benchmarks, ascorbic acid and butylated hydroxytoluene (BHT), which present IC_50_ values of 0.05 and 0.13 mg mL^−1^ (ABTS) and of 0.04 and 0.28 mg mL^−1^ (DPPH), respectively [5], we can conclude that grape seeds are, indeed, a relevant source of antioxidant compounds. Impressively, the GSEs, which are complex mixtures of different compounds, presented similar antioxidant capacity to the tested benchmarks, which are pure compounds. Previous studies have reported the potential of natural plant-derived compounds, including grapes, as antioxidant agents for cosmetic/skincare applications [40,41].

#### 3.1.2. Antimicrobial Activity

Both GSEs were also tested for their antimicrobial potential against skin microbiota commensal bacteria, such as *S. epidermidis*, methicillin-sensitive *S. aureus* (MSSA), methicillin-resistant *S. aureus* (MRSA), and *C. acnes*. The extracts were tested at the following concentrations: 50, 25, 12.5, 6.25, 3.13, and 1.56 mg mL^−1^ for MBC determination. Minimum inhibitory concentrations (MICs) could not be evaluated since the extracts precipitated, making it impossible to assess the turbidity of the medium caused by microbial growth. Therefore, only the MBC results were evaluated, which are presented in Table 2. From the tested microorganisms, only *C. acnes* was not inhibited by any of the extracts at the tested concentrations. Moreover, *S. epidermidis*, commonly found on human skin, was only inhibited by the highest tested concentration (50 mg mL^−1^) of both GSEs. However, this concentration is quite high and, therefore, it is not considered for product incorporation since, as discussed ahead (Section 3.6.1), this concentration was found to be toxic for skin cells. The non-inhibition of these bacteria can be seen as a positive outcome since, in balance, these bacteria are important for skin homeostasis maintenance, host defence, and innate immune response [42]. Furthermore, *S. epidermidis* is capable of inhibiting the adhesion of virulent *S. aureus* strains [42,43]. This is relevant since, as discussed before, *S. aureus* has been associated with exacerbating skin diseases, such as atopic dermatitis. That said, the obtained results for *S. aureus* inhibition are promising and may lead the way towards the treatment and/or prevention of skin conditions. Comparing both extracts, the results were similar, except for MSSA, where GSE-Ov showed higher antimicrobial capacity.

#### 3.1.3. Individual Phenolic Compound Identification by HPLC

**Figure 3:**
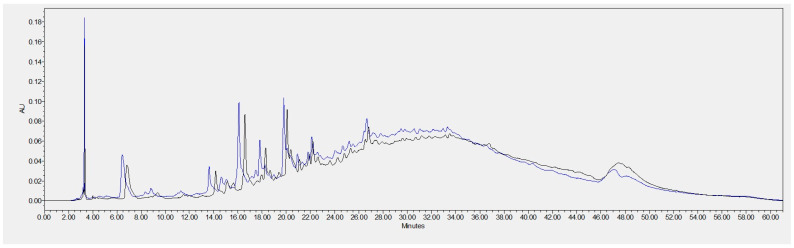
Chromatogram of the HPLC analysis of the GSEs and the respective identified phenolic compounds at 280 nm (blue: GSE-Sv; black: GSE-Ov).

As discussed earlier, phenolic compounds are secondary plant metabolites (e.g., derived from grapevines) that exhibit several biological properties of relevance, such as antimicrobial, antioxidant, and anti-inflammatory activities; therefore, they show great potential for cosmetic applications. In this line, both GSEs were analysed by HPLC for individual polyphenol identification. In total, four different compounds were identified from the chromatograms (Figure 3). The most predominant compounds were those at a wavelength of 280 nm; therefore, the remaining wavelengths studied (320, 360 and 528 nm) are presented in Supplementary Figure S1.

We identified gallic acid, catechin, procyanidin B1, and procyanidin B2. The latter was only found in GSE-Sv, as reported in Table 3. All the identified phenolic compounds are normally found in grape seed extracts (e.g., Pinot Noir, Cabernet Sauvignon, Marselan, Tamyanka) [44]. Regarding gallic acid and catechin, they were found in higher quantities in GSE-Sv, as revealed by the peak areas (Table 3). Apart from the procyanidin B1 case, these findings are not consistent with the results of antioxidant activity, where GSE-Ov showed higher total phenolic content and radical scavenging activity. The comparison of area values is warranted since both extracts were analysed at the same concentration. Besides their known antioxidant potential, these compounds are also reported to be beneficial for skincare applications. For example, gallic acid has been suggested as a candidate for the prevention of UVB-induced premature skin ageing by decreasing skin dryness, thickness, and wrinkle formation. Also, IL-6 production was suppressed (anti-inflammation) and type-I procollagen was stimulated [45]. Another study showed that gallic acid inhibited melanin production by downregulating tyrosinase activity and was capable of acting as a skin-whitening agent [46]. Furthermore, (+)-catechin has been found to inhibit the inflammatory mediator prostaglandin E_2_, tumour necrosis factor-alpha (TNF-α), and proinflammatory cytokines IL-1β and IL-6 [47]. Regarding procyanidins, the anti-inflammatory activity of procyanidin B2 has also been reported through the inhibition of NF-kB, IL-6, and pro-IL-1β [48]. Lastly, both procyanidins (B1 and B2) have shown moderate inhibitory effects over collagenase and elastase activities, although in a dose-dependent manner [49]. By analysing the chromatogram, it can be concluded that there is a more pronounced profile of GSE-Sv phenolic compounds. However, it is possible that the compounds presented in Table 3 are not fully representative of the antioxidant potential of the extracts. That is, the potential of GSE-Ov verified by the antioxidant activity results could be due to other possibly more antioxidant phenolic compounds that are present but not identified, e.g., hydroxycinnamic acids (Supplementary Figure S2).

#### 3.1.4. Fourier Transform Infrared Analysis

**Figure 4:**
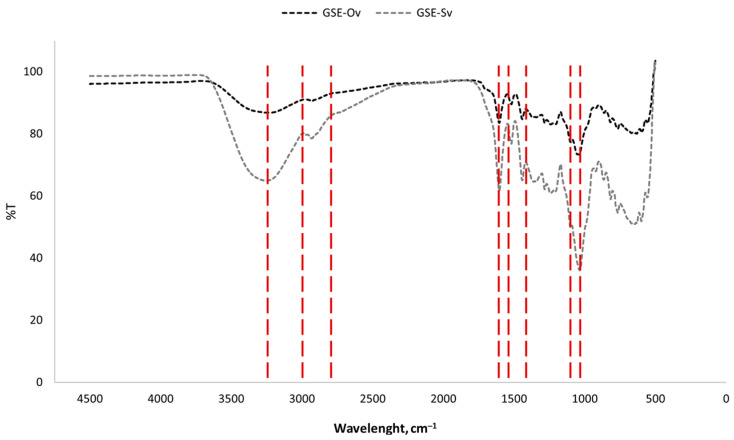
FTIR spectra of the GSEs. Red lines indicate the wavelengths of the functional groups, discussed below.

Under Fourier Transform Infrared (FTIR) spectroscopy, each substance has a unique spectrum fingerprint that differentiates it from other compounds [50]. The results of the FTIR analysis of the GSEs are presented in Figure 4.

We can observe that both GSEs have identical spectra and, therefore, share the main functional groups. It was possible to distinguish several peaks/bands that represent functional groups and modes of vibration and the components to which they are related. The broad band peaking at approximately 3250 cm^−1^ represents a stretching O-H bond, which may be due to the presence of phenolic compounds (rich in hydroxyl groups), polysaccharides, and lignins. The zone between around 2800 and 3000 cm^−1^, which seems to be composed of two unresolved peaks, corresponds to asymmetric and symmetric stretching bonds of CH_2_ groups derived from lignins and lipids. The peak around 1600 cm^−1^ is relative to COO^−^ groups and aromatic C=C bonds found in pectins and phenolics. The two peaks around 1500 cm^−1^ correspond to aromatic stretching C-C bonds from phenolic compounds. The peak around 1150 cm^−1^ is due to the aromatic stretching of C-H bonds from phenolics, and around 1000 cm^−1^, there is a peak relative to the stretching of C-O and C-C bonds from polysaccharides and pectins. This spectra analysis is in accordance with Günter and Popeyko [51], Nogales-Bueno et al. [52], and Lucarini et al. [53], and suggests that the extraction process was efficient.

### 3.2. GSE-Ov Encapsulation Microdispersion

Advanced delivery systems, at the nano- and/or micro-scales, offer several advantages for the administration of specific compounds. These personalized systems protect the encapsulated compounds, preventing their degradation, and can transport and release them more effectively on the targeted tissues. Herein, microdispersions are the chosen system because of their lipid character and their perfect interaction with the skin and its ceramides, which are one of the main groups of skin lipids and play a vital role in its barrier function [54]. For these reasons, we aimed to develop a microdispersion system capable of efficiently entrapping and delivering GSE-Ov, the extract that demonstrated the best phenolic compound profile and biological properties, to the skin.

### 3.3. Optimization of the Encapsulation Process

To optimize the microdispersion preparation, several tests were carried out considering the Zeta potential (ζ) and Polydispersity Index (PDI) as fundamental parameters for the development of the system (Table 4). In relation to these two measurements, ζ is better at a higher absolute value, which represents a greater charge and, therefore, a greater repulsion between the particles and consequent stability over time. A lower PDI value represents greater microdispersion size homogeneity. The lower the PDI, the lower the index of particle subpopulations [55,56]. For this purpose, the following microdispersion production methods were tested: (I) Lipid Film Hydration (LFH) and (II) Reverse-Phase Evaporation (RPE). LFH obtained better ζ and PDI results and, therefore, was the method chosen to carry out the investigation. This method (LFH) is often preferred in laboratory settings as it is simple and does not require specialized equipment [36].

Once the method was chosen, condition III consisted of adding the extract to the microdispersions, which resulted in a relatively unstable and prone-to-agglomerate formation mixture (decreased ζ value). To solve this issue, for condition IV, chitosan was added as a microdispersion coat [57,58], leading to the intended increase in the ζ value from −5.1 to −9.3 mV, which was not that significant. However, the PDI value increased from 0.405 to 1.000, which was not intended. Moreover, chitosan precipitated, which was also not desired. So, for condition V, chitosan was replaced with pectin [57] and, indeed, the ζ value improved significantly to −20.3 mV, and the uniformity index (similar to the PDI) was 0.637. Pectin was chosen as the biopolymer, as it is found in grape pomace [59], and in future studies, pectin itself can be extracted and used from the same pomace. No sonication step was needed because the measurement equipment provided ultrasounds of its own. In this line, the final formulation consisted of the LFH method for the GSE-Ov-loaded microdispersion coated with pectin.

### 3.4. Characterization of the Selected Microdispersion Preparation

#### 3.4.1. Zeta Potential, Size Distribution, and Morphology

After optimizing the production process, some physical tests were carried out on the selected microdispersions. The first evaluated feature was the zeta potential (ζ), and the obtained value was −20.3 mV. This suggests that the developed system is stable, with no tendency to agglomerate over time since its charge is close to that reported in the literature (ζ~± 30 mV), which is ideal for long-term stability [60]. The negative electrical surface charge was due to the presence of lecithin [61,62], and pectin was used to increase stability.

**Figure 5:**
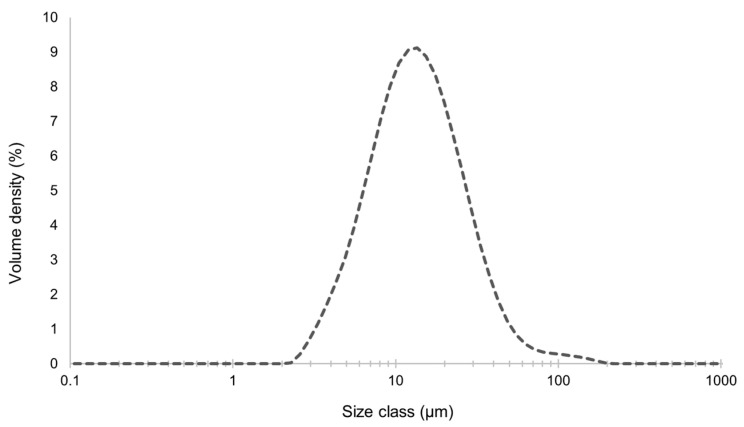
Size distribution of the GSE-Ov-loaded microdispersion.

Another assessed characteristic was the size distribution. This variable is relevant since it directly impacts the cellular uptake, transportation, and accumulation behaviour of the microdispersion [63]. The results presented in Figure 5 indicated that the microdispersion had a mean diameter of 13.6 µm, which meets the micro-scale range, as desired. This average size is appropriate for topical applications, such as on the skin [28]. In addition, the empty microdispersions had a mean diameter of 4.87 µm. Microdispersions on the micro-scale have some advantages, such as higher encapsulation efficiency with higher microdispersion size [64], namely, relative to large biomolecules, and the fact that they avoid nano-bioaccumulation issues and related legislation restrictions (labelling, etc.) [23]. Furthermore, a uniformity size measure (similar to PDI) of 0.637 was obtained, which indicates some size heterogeneity but is still acceptable [55]. The PDI is close to 0.5, which indicates a prevalence of sizes close to 15 µm, in this case.

**Figure 6:**
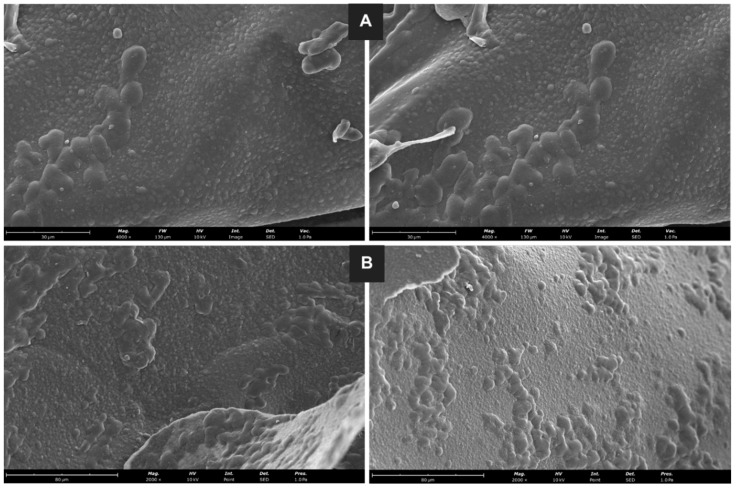
SEM images of the powdered ((**A**) magnification of 4000×) and splintered ((**B**) magnification of 2000×) lyophilized GSE-Ov-loaded microdispersions.

For microdispersion morphology, a scanning electron microscopy (SEM) analysis was performed, and the images are presented in Figure 6. In all pictures, near spherical-shaped and regular microdispersion particles can be seen, in addition to some agglomerates. Also, some size heterogeneity is observed, associated with the microdispersion development process. The agglomerates seem to be formed out of larger microdispersion particles. Also, it has been reported that sample preparation for SEM analysis may be responsible for the formation of some agglomerates [65]. The results were similar for the powdered (Figure 6A) and splintered (Figure 6B) lyophilized GSE-Ov-loaded microdispersions.

#### 3.4.2. FTIR Analysis

**Figure 7:**
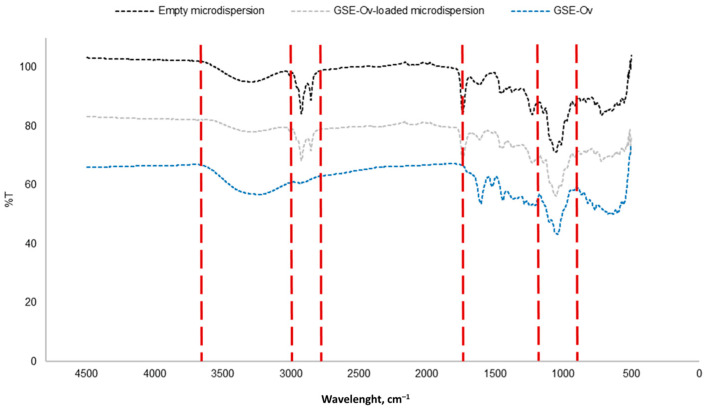
FTIR spectra of GSE-OV and the empty and GSE-OV-loaded microdispersions. Red lines indicate the wavelengths of the functional groups, discussed below.

An FTIR analysis was performed for the empty and GSE-Ov-loaded microdispersions. The resultant spectra are presented in Figure 7.

Analysing the spectra, the first conclusion that can be made is that both the empty and loaded microdispersions present a similar fingerprint. Importantly, the lack of shifts in the identified bands reveals that the encapsulation process neither alters the structure of compounds in GSE nor promotes new bond formation, as desired. The functional group were not, therefore, compromised by the encapsulation process, as confirmed by the biological properties described below. Since the main component of the microdispersions is soy lecithin, there is a group of bands/peaks that are characteristic of this substance. For instance, the region between 2800 and 3000 cm^−1^, composed of two peaks, corresponds to asymmetric and symmetric stretching bonds of CH_2_ groups. The peak around 1750 cm^−1^ corresponds to stretching C=O bonds from aliphatic ester groups. The peaks in the zone approximately between 950 and 1200 cm^−1^ refer to stretching vibrations of P=O, P-O-C, and C-O-C groups. Similar results can be found regarding the lack of interaction between lecithin as an encapsulant and other encapsulated compounds (e.g., tea polyphenol). Besides lecithin, pectin was used for microdispersion particle-coating and is responsible for the broadband approximately between 3000 and 3600 cm^−1^, which corresponds to stretching O-H groups from intra- and intermolecular hydrogen bonds related to galacturonic acid. This spectra analysis was performed according to Merino et al. [66], Santos et al. [67], Whittinghill et al. [68], and Pezeshky et al. [69].

### 3.5. Encapsulation Efficiency and Phenolic Compound Release Profile

The selected microdispersion presented an encapsulation efficiency of 88.8%, which, in comparison with literature values (ranging from 52 to 91% [23]), is considered a very positive result. The phenolic compounds released from the microdispersion were also assessed, and the results are presented in Table 5. The assays were carried out for 24 h, and the maximum percentage of phenolic compounds released was reached with 59.4% of the initial content. Within the first 6 h, the release profile was nearly linear, with almost 50% of the phenolic compounds being in solution at this time, and slowed down considerably onwards. Interestingly, a study by Lu et al. [70] reported a similar release profile of phenolic compounds from GSE entrapped in a microdispersion, at this time. Moreover, in another study, Gibis et al. [71] obtained 55.5% release within 24 h, which also resembles the presented result.

Here, it can be concluded that the systems revealed a prolonged release over time. However, being an in vitro study, real application parameters, such as topical placement, mechanical abrasion, and/or enzymatic action were not considered [72,73]. In this way, it is expected that in vivo release will be higher and faster in time. Nevertheless, these results appear promising for a topical delivery system.

### 3.6. Safety and Skincare Potential of Encapsulated GSE-Ov

After physicochemical characterization, GSE-Ov, the GSE-Ov-loaded microdispersion, and the empty microdispersion were tested for their skincare potential, including a safety assay and the assessment of their anti-inflammatory activity and capacity to increase pro-collagen I α1 production.

#### 3.6.1. Cytotoxicity

**Figure 8:**
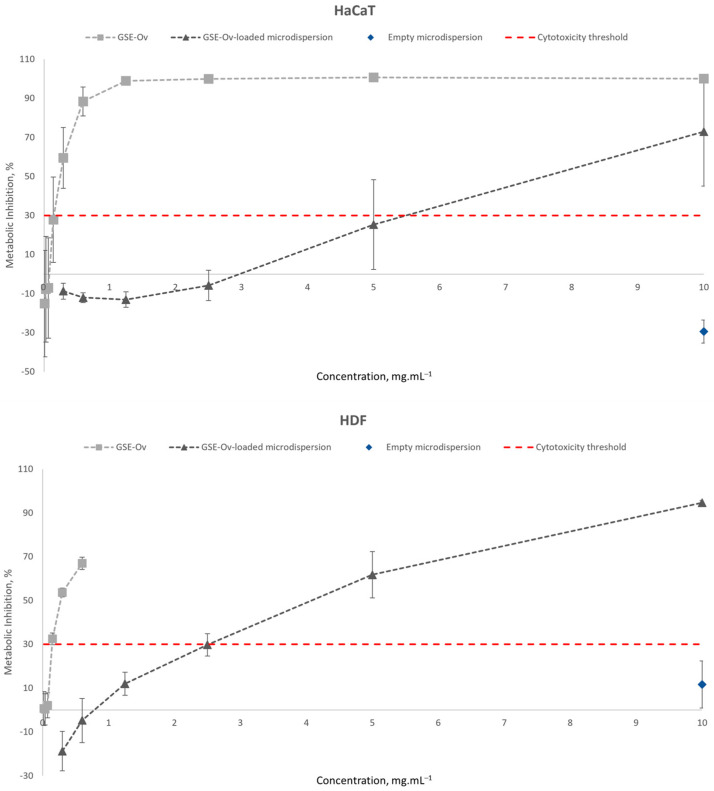
Cytotoxicity study of GSE-Ov, the GSE-Ov-loaded microdispersion, and the empty microdispersion on the HaCaT and HDF cell lines.

Regarding their safety, these samples were tested on the HaCaT and HDF cell lines, and the results are presented in Figure 8. GSE-Ov was safe for concentrations below ~0.15 mg mL^−1^ for the HaCaT and HDF cell lines, reaching metabolic inhibitions of 27.9 and 32.5%, respectively. Concerning the GSE-Ov-loaded microdispersion, it was shown to be safe up to concentrations of approximately 5 mg mL^−1^ for HaCaT and 2.5 mg mL^−1^ for HDF cells, with metabolic inhibitions of 25.4 and 29.8%, respectively. According to this, microdispersion application seems to be favourable, highlighting the importance of the GSE-Ov encapsulation, since the extract alone was shown to be significantly more cytotoxic. Even so, the results of the antioxidant activities are compatible with these since the obtained IC_50_ values are within the safe range for both cells. Finally, for both cell lines, the empty microdispersion was shown to be non-cytotoxic in the maximum tested concentration (10 mg mL^−1^), with metabolic inhibitions of −29.4 and 11.6% for HaCaT and HDF, respectively. In all cases, HDF cells were shown to be more sensitive to the tested extracts. Similar results were reported by Duarte et al. [5]. We note that negative inhibition values may result from an increase in cellular proliferation [74], i.e., in these cases, there may have been an over-metabolization of the PrestoBlue™ reagent caused by the samples and, therefore, an emission of fluorescence superior even to the positive controls.

#### 3.6.2. Anti-Inflammatory Activity

**Figure 9:**
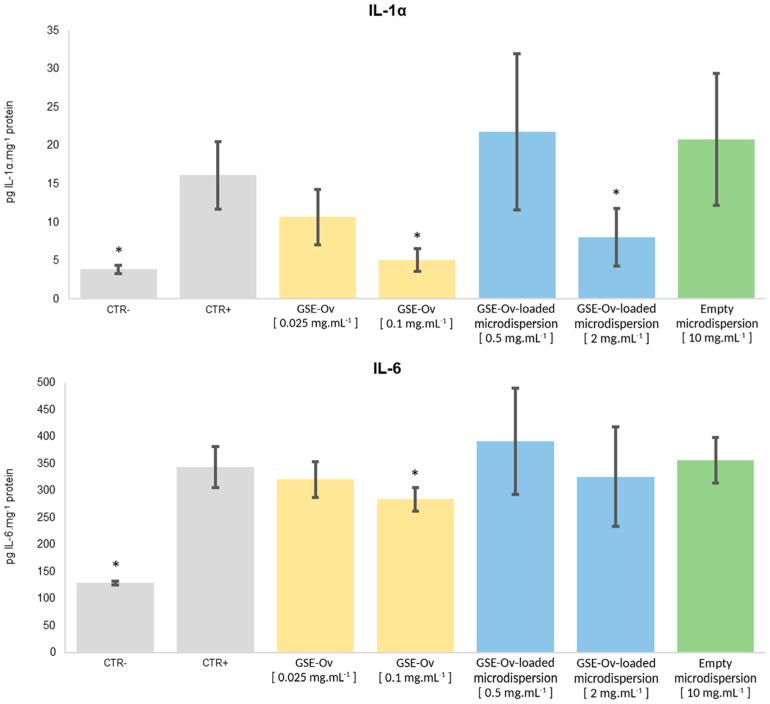
Effect of GSE-Ov, the GSE-Ov-loaded microdispersion, and the empty microdispersion on keratinocytes (HaCaT) by assessing pro-inflammatory cytokine IL-1α and IL-6 levels under an inflammatory stimulus (pollution particles). Mean values (solid bars) are expressed as pg cytokine mg^−1^ total protein, and standard deviations are represented by bars; * significantly different from positive control (CTR+) (*p* < 0.05).

As discussed before, inflammation plays a major role in some skin diseases; therefore, it is important to evaluate the anti-inflammatory potential of the extracts. For this purpose, the effect of GSE-Ov, the GSE-Ov-loaded microdispersion, and the empty microdispersion on the production of pro-inflammatory cytokines (IL-1α and IL-6) by keratinocytes exposed to urban air dust particles were measured, and the results are presented in Figure 9. All samples were tested according to their safe concentration range.

Regarding IL-1α, its overexpression has been associated with symptom exacerbation and disease progression in psoriasis, atopic dermatitis, and neutrophilic dermatoses (e.g., hidradenitis supurrativa). Nonetheless, this interleukin has important functions in the skin. For example, in psoriasis and atopic dermatitis lesions, keratinocytes extensively produce IL-1α, which has been suggested as a skin disease biomarker and is capable of helping to maintain skin integrity and barrier [75,76,77]. Concerning the anti-inflammatory potential of the samples, GSE-Ov was able to reduce IL-1α production in a concentration-dependent manner. In the case of 0.1 mg mL^−1^ GSE-Ov, the amount of cytokine produced decreased from 16.1 to 5.1 pg IL-1α mg^−1^ of protein (*p* < 0.05). For the GSE-Ov-loaded microdispersion, only the most concentrated samples (2 mg mL^−1^) were able to decrease the amount of cytokine (from 16.1 to 8.1 pg IL-1α mg^−1^ of protein, *p* < 0.05). Such an effect could be due to the encapsulation of the GSE, leading to higher protection and, therefore, decreased immediate bioavailability. For a more complete evaluation, this study should be conducted over time. Finally, the empty microdispersion did not exert any anti-inflammatory activity over IL-1α production, as expected.

On the other hand, IL-6 is produced by many different types of cells and is expressed under many states of cellular stress, including inflammation, infection, wound sites, and cancer [78]. None of the tested samples presented significant anti-inflammatory activity over IL-6 production, although a slight reduction in cytokine amount was observed for GSE-Ov at 0.1 mg mL^−1^ (*p* < 0.05). Interestingly, a study by Nallathambi et al. [79] reported that GSE was able to reduce IL-1α and IL-6 expression. However, this was carried out with intestinal cellular models, and, regarding IL-6, the data showed a smaller expression than that found in our essay. In our work, it is possible that the tested GSE-Ov concentration was not enough to induce such effects. Similar to the GSE-Ov composition, catechin and procyanidin B1 were also detected in the mentioned extract. To better understand the anti-inflammatory effect of the extract, studies on other cytokines could be conducted. Nevertheless, the results show some specificity of the extract towards skincare since it inhibits IL-1α, which is a cytokine specifically linked to skin inflammation. On the other hand, IL-6 is a more systemic cytokine. 

#### 3.6.3. Human Pro-Collagen I α1 Production

**Figure 10:**
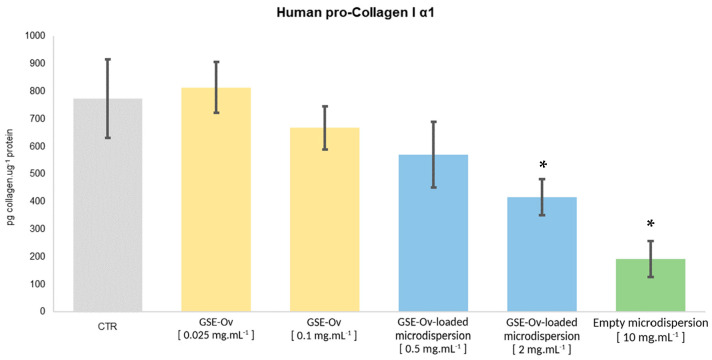
Effect of GSE-OV, the GSE-Ov-loaded microdispersion, and the empty microdispersion on the production of pro-Collagen I α1 by HDF cells. Mean values (solid bars) are expressed as pg collagen µg^−1^ protein and the standard deviation is represented by bars; * significantly different from the control (CTR) (*p* < 0.05).

Collagen is the most abundant component of the extracellular matrix (ECM) and the skin’s principal structural protein [80,81]. It is responsible for structure, stability, and strength, particularly within the dermal layers [82], and it plays an important role in preventing skin ageing. Decreasing collagen density has been linked to the process of skin ageing, particularly when related to sun exposure (photoaging), leading to loss of skin integrity and elasticity [82,83]. For these reasons, the effect of GSE-Ov, the GSE-Ov-loaded microdispersion, and the empty microdispersion on Human pro-Collagen I α1 production by HDF was assessed, and the results are presented in Figure 10. At 10 mg mL^−1^, the empty microdispersion inhibited collagen production, reducing it from 773.32 to 191.46 pg collagen ug^−1^ protein (*p* < 0.05). Interestingly, lecithin, the main component of the microdispersion formulation, has been reported to exert negative effects on collagen, like decreased accumulation (by stimulating collagenase activity) and gene expression [84,85]. These findings could be relevant for improving the GSE-Ov encapsulation process and selecting the right concentration of this material. Moreover, at both concentrations, the GSE-Ov-loaded microdispersion also inhibited some collagen production, with the most concentrated sample exerting higher inhibition. Concerning GSE-Ov, at 0.025 mg mL^−1^, it stimulated the average production of collagen by 5.13%, although with no significant differences compared to the control (*p* > 0.05). Nevertheless, GSE has been reported to promote collagen fibre deposition and strengthen collagen-based tissues [86]. Likewise, it could be beneficial to conduct studies on the extracellular deposition of collagen and the impact of the extract on the production of other extracellular matrix components, e.g., elastin and fibronectin.

## 4. Conclusions

The objectives of this work were the production and characterization of a GSE for potential skincare and cosmetic applications. To achieve these objectives, two different GSEs were studied. Based on their antioxidant and antimicrobial activities, the more promising extract was GSE-Ov. The antioxidant results showed a huge radical scavenging potential, while the inhibition of *S. aureus* strains (MRSA and MSSA), together with the non-inhibition of *S. epidermidis*, suggested beneficial effects on several skin pathologies. Furthermore, we aimed to use an encapsulation technique in order to promote GSE stability. The microdispersion provided a stable and size-appropriate delivery system. In this sense, the soy lecithin microdispersion coated with pectin was the best formulation, presenting an encapsulation efficiency of 88.8% and a controlled release of phenolic compounds over several hours.

Regarding safety, the GSE-Ov-loaded microdispersion was shown to be safe below 5 and 2.5 mg mL^−1^ for HaCaT and HDF cells; therefore, the sample could be used as an antioxidant ingredient since the DPPH IC_50_ value falls within the safe range. Furthermore, the extract showed some potential as a skincare ingredient, as it also downregulated IL-1α production.

In summary, it is believed that the use of GSE in skincare holds great potential, especially when delivered in microdispersions.

## Figures and Tables

**Table 1:** Total phenolic content and antioxidant activity values (mean ± SD) determined by the Folin–Ciocalteau method and ABTS and DPPH assays for both GSEs and the two antioxidant benchmarks (ascorbic acid and butylated hydroxytoluene (BHT)).

Analysis	GSE-Ov	GSE-Sv	Ascorbic Acid	BHT
Total phenolics (mg GA eq g^−1^ dry extract)	258.96 ± 30.94 ^a^	203.72 ± 42.64	---	---
ABTS IC_50_ (mg mL^−1^)	0.138 ± 0.034 ^a^	0.163 ± 0.015	0.05 *	0.13 *
DPPH IC_50_ (mg mL^−1^)	0.079 ± 0.003 ^a^	0.088 ± 0.004	0.04 *	0.28 *

^a^ significantly different from GSE-Sv (*p* < 0.05) within a given row; * values retrieved from Duarte et al. [5].

**Table 2:** GSE antimicrobial activity against *Staphylococcus epidermidis*, methicillin-sensitive *S. aureus* (MSSA), methicillin-resistant *S. aureus* (MRSA), and *Cutibacterium acnes*.

Microorganism	GSE-Ov	GSE-Sv
	MBC mg mL^−1^	
*S. epidermidis*	50	50
Methicillin-sensitive *S. aureus* (MSSA)	3.125	6.25
Methicillin-resistant *S. aureus* (MRSA)	6.25	6.25
*C. acnes*	>50	>50

**Table 3:** Retention times and peak areas (As) of phenolic compounds identified in both GSEs by HPLC at 280 nm.

Polyphenol	Retention Time (min)	GSE-Ov (A1)	GSE-Sv (A2)	A1/A2
Gallic acid	6.834	827,724	1,059,774	0.78
Catechin	16.579	1,111,979	1,333,777	0.83
Procyanidin B1	14.164	339,778	225,966	1.50
Procyanidin B2	17.569	---	572,197	---

**Table 4:** Optimization of the microdispersion production: values of the zeta potential (ζ) and PDI/Uniformity (mean ± SD) for each condition.

Conditions #	Process Description	ζ (mV)	PDI/Uniformity
I	LFH + 1 min sonication (70% amplitude)	−29.7 ± 2.9	0.407 ± 0.008
II	RPE (Reverse Phase Evaporation) method	−14.4 ± 1.3	0.432 ± 0.031
III	Condition #I + GSE-Ov	−5.1 ± 1.1	0.405 ± 0.014
IV	Condition #III + chitosan	−9.3 ± 0.7	1.000 ± 0.000
V (final)	LFH + GSE-Ov + pectin	−20.3 ± 2.4	0.637 *

* Uniformity was measured instead of PDI.

**Table 5:** Phenolic compound release profile by the microdispersion.

Time (h)	Percentage of Released GSE (Mean ± SD)
1	12.67 ± 0.46%
2	24.95 ± 0.69%
4	37.84 ± 0.40%
6	48.18 ± 0.75%
24	59.42 ± 0.41%

## Data Availability

No data were reported in this study.

## Supplementary Materials

The following supporting information can be downloaded at: https://www.mdpi.com/article/10.3390/molecules29163717/s1, Figure S1: Chromatogram of the HPLC analysis of the GSEs and respective identified phenolic compounds at black: 280 nm, dark blue: 320 nm, green: 360 nm, light blue: 528 nm; Figure S2: Chromatogram of the HPLC analysis of the GSEs and respective identified phenolic compounds at 320 nm (blue: GSE-Sv; black: GSE-Ov).

## Abbreviations

ABTS, 2,20-azinobis (3-ethylbenzothiazoline-6-sulfonic acid); CTRL, control; CTRL+, positive control; DAD, Diode Array Detector; dH_2_O, deionized water; DMEM, Dulbecco’s Modified Eagle Medium; DMSO, dimethyl sulfoxide; DPPH, 2,2-diphenyl-1-picrylhydrazyl; EE, encapsulation efficiency; FBS, foetal bovine serum; FTIR, Fourier-Transform Infrared; GSE-Ov, grape seed extract—one variety; GSE-Sv, grape seed extract—several varieties; HaCaT, immortalized human keratinocytes; HDFs, human dermal fibroblasts; HPLC, High-Performance Liquid Chromatography; IL-1α, Interleukin 1α; IL-6, Interleukin 6; MBC, minimum bactericidal concentration; MH, Mueller Hinton; MIC, minimum inhibitory concentration; MRSA, methicillin-resistant *S. aureus*; MSSA, methicillin-susceptible *S. aureus*; TFA, trifluoroacetic acid; TS, tryptic soy.
