# Supplementary material for: Elevating Skincare Science: Grape Seed Extract Encapsulation for Dermatological Care
Source: Molecules. 2024 Aug 6;29(16):3717. doi: 10.3390/molecules29163717 (PMC11357433; doi:10.3390/molecules29163717)
Supplement: Supplementary file 1 [file molecules-29-03717-s001.zip › molecules-3111477-supplementary.pdf]

GSE-Ov

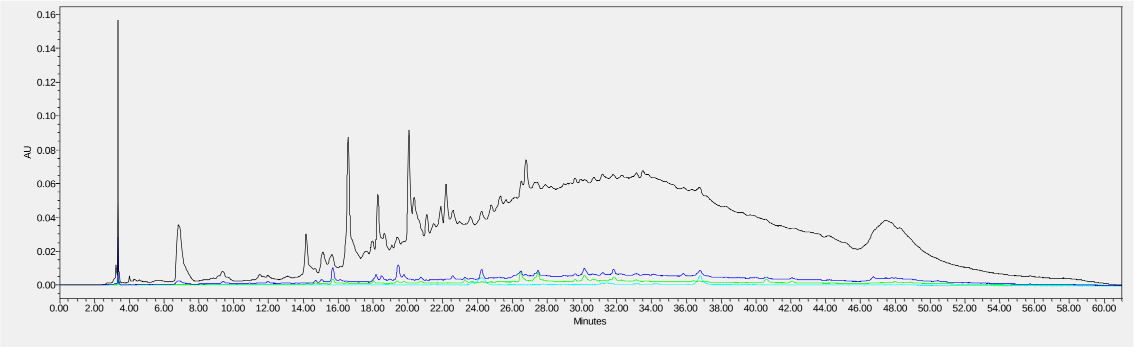


GSE-Sv

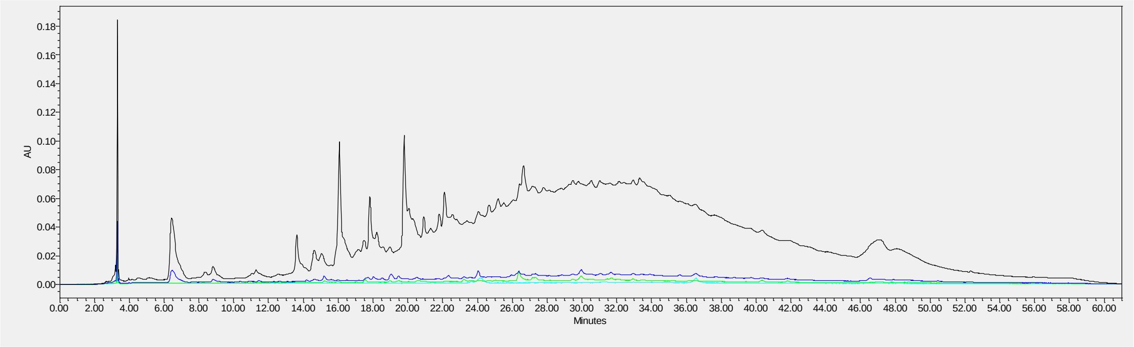


*Figure S1* - Chromatogram of the HPLC analysis of the GSEs and respective identified phenolic compounds at black: 280 nm, dark blue: 320 nm, green: 360 nm, light blue: 528 nm.

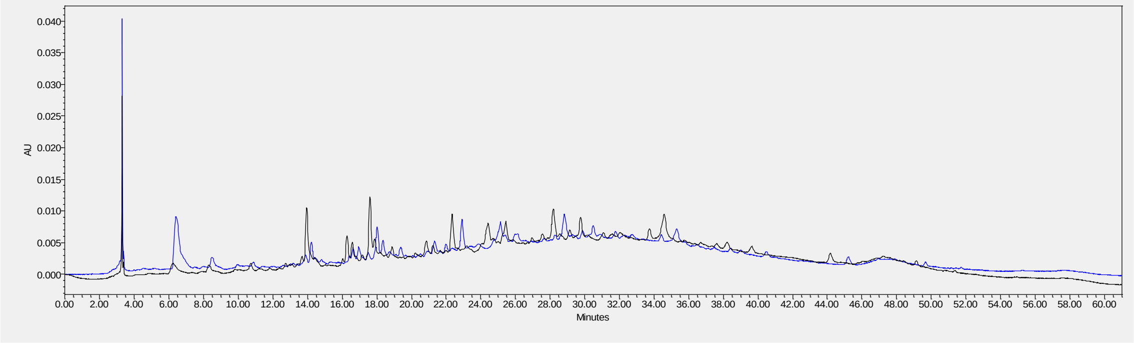


*Figure S2* - Chromatogram of the HPLC analysis of the GSEs and respective identified phenolic compounds at 320 nm (blue: GSE-Sv; black: GSE-Ov).
